# Growth and morphological analysis of segmented AuAg alloy nanowires created by pulsed electrodeposition in ion-track etched membranes

**DOI:** 10.3762/bjnano.6.131

**Published:** 2015-06-08

**Authors:** Ina Schubert, Loic Burr, Christina Trautmann, Maria Eugenia Toimil-Molares

**Affiliations:** 1 Materials Research Department, GSI Helmholtzzentrum für Schwerionenforschung GmbH, Darmstadt, Germany; 2 Department of Materials- and Geo-Science, Technische Universität Darmstadt, Darmstadt, Germany

**Keywords:** AuAg alloy, cyclic voltammetry, electrodeposition, ion-track technology, nanogaps, segmented nanowires

## Abstract

**Background:** Multicomponent heterostructure nanowires and nanogaps are of great interest for applications in sensorics. Pulsed electrodeposition in ion-track etched polymer templates is a suitable method to synthesise segmented nanowires with segments consisting of two different types of materials. For a well-controlled synthesis process, detailed analysis of the deposition parameters and the size-distribution of the segmented wires is crucial.

**Results:** The fabrication of electrodeposited AuAg alloy nanowires and segmented Au-rich/Ag-rich/Au-rich nanowires with controlled composition and segment length in ion-track etched polymer templates was developed. Detailed analysis by cyclic voltammetry in ion-track membranes, energy-dispersive X-ray spectroscopy and scanning electron microscopy was performed to determine the dependency between the chosen potential and the segment composition. Additionally, we have dissolved the middle Ag-rich segments in order to create small nanogaps with controlled gap sizes. Annealing of the created structures allows us to influence their morphology.

**Conclusion:** AuAg alloy nanowires, segmented wires and nanogaps with controlled composition and size can be synthesised by electrodeposition in membranes, and are ideal model systems for investigation of surface plasmons.

## Introduction

The synthesis of multicomponent heterostructure nanowires is currently being intensively investigated. It has become evident that the combination of several materials in one nanostructure gives rise to specific functionalities that are not exhibited by the individual single components [1–4]. Different types of heterostructures such as core–shell, axially segmented or alloy nanowires are being developed and characterized. Their applications in the fields of optics [5–8], magnetism [9–10], electronics [11–12] and solar harvesting [13–15] are envisaged. The functionality of the multicomponent nanostructures is determined by their properties including composition, dimension, and crystallinity. Therefore, to develop synthesis methods that guarantee a precise control of these properties is important.

A powerful technique to synthesise nanowires with well-controlled morphological and crystallographic characteristics is the so-called template method [16–17]. A large number of wires up to 10^10^ cm^−2^ can be grown simultaneously. In particular, in the case of polymer templates created by ion irradiation and chemical track-etching, nanowires with various shapes such as cylindrical, conical and biconical, with lengths between 1 and 100 μm, and diameters as small as about 15 nm can be fabricated [18]. By choosing different types of polymer membranes the surface morphology of the nanowires can be varied [19–20]. Since the successful growth of Cu/Co and Ni/Cu multilayer nanowires back in the 1990s [1–2,21], the template method has allowed for the growth of many different segmented structures combining polymers, semiconductors, and metals, such as Au–TiO_2_ [11], Au–polypyrrole [22], Cu–Se [23], and Au–Co [24]. While segmented nanowires can be grown by sequential exchange of the electrolyte [8,25–26], it is also possible to use a single electrolyte and control the composition of the segments by tuning reduction potential and electrolyte composition [27–30]. The segment lengths are adjusted by the amplitude and length of the applied pulse [27,30–31]. Being able to fabricate a very large amount of wires with excellent control over segment length is crucial for many applications [28,30,32–33].

AuAg nanowires are particularly investigated in the fields of optics and electronics. Bimetallic AuAg nanowires are very promising as sensing tools for surface enhanced Raman spectroscopy [34]. As an example, AuAg alloy nanorods show enhanced sensing resolution compared to pure Au nanowires [35] while Au@Ag core shell nanorods allow to adjust the resonance frequency by varying the shell thickness [36]. Furthermore, optical applications include the readout of striping patterns in AuAg segmented nanowires via optical brightfield microscopy [37] and fluorescence spectroscopy for bioanalysis, such as biological multiplexing [7]. Finally, by etching one elemental type of segments in metallic barcoded wires, small gaps separating the unetched (remaining) segments are being created [38–40], which could find applications as hot spots for surface enhanced Raman spectroscopy [38,41–43] and for biosensing [44]. Furthermore, such gaps with precisely controlled dimensions allow for the systematic investigation of multipole surface plasmon modes [39,45–46]. Because of their high electrical conductivity [47], Au and Ag metallic wires have also great potential as electronic components [7] and, thus, Au nanowires separated by small gaps are very promising as nanowire electrodes that can be used as field effect transistors [48] and for the capture and electrical characterization of nanoparticles [49]. For all these applications, the length of the segments plays a determining role and, thus, the length distribution achieved during the simultaneous growth of nanowire arrays should be analysed and discussed in detail.

In this work, we synthesise segmented AuAg alloy nanowires by pulsed electrodeposition in track-etched membranes using a single electrolyte. In particular, we aim at controlling all segment sizes. We analyze the segment size distribution in detail and discuss the deposition conditions needed to fabricate very small nanogaps by subsequent etching of the middle Ag-rich segments in nitric acid. We apply cyclic voltammetry and analyze the deposition process in the channels of ion-track etched membranes compared to cyclic voltammetry for macroelectrodes.

## Experimental

Polycarbonate foils (Makrofol N, Bayer AG) with a thickness of 30 μm were irradiated with Au ions (ca. 2 GeV) at the linear accelerator UNILAC at GSI Helmholtzzentrum für Schwerionenforschung. Each ion crossing the foil creates a damage trail, the so-called ion track. A fluence of 10^9^ ions per cm^2^ was applied. By chemical etching, the damaged material is selectively dissolved converting the track into an open nanochannel [50]. The etching time controls the diameter of the resulting pores. Here, the irradiated foils were etched for 5 min in a 6 M NaOH solution at 50 °C to fabricate channels with diameters of about 110 nm. A low resolution and a high resolution SEM image of such an ion-track etched membrane are depicted in Figure S1 of Supporting Information File 1.

In a next step, a Au layer was sputtered on one side of the polymer template, serving as the cathode for the deposition. It was reinforced with Cu, electrodeposited at room temperature in a two electrode set-up at a potential of −0.5 V between the Cu anode and cathode. The electrodeposition of nanowires in the pores of the template was performed in a three-electrode set-up using a potentiostat (GAMRY Instruments, Reference 600TM) and a platinum wire as counter electrode. All potentials given here are reported versus the reference electrode, being Ag/AgCl (sat. KCl). Before starting the deposition, the electrolyte in the electrochemical cell was preheated to 60 °C. For wire deposition and cyclic voltammetry, we used basic cyanide electrolytes (pH 13). They contain 0.25 M Na_2_CO_3_ (*>*99.8%, Carl Roth), and KAu(CN)_2_ (Carl Roth) and KAg(CN)_2_ (Sigma Aldrich) in different concentrations as given in the results and discussion section [51].

Cyclic voltammograms (CVs) were recorded applying a scan rate of 30 mV/s and a step size of 5 mV. Counter electrode and reference electrode are the same as given above. The geometric area of the membrane used for deposition and the CVs was about 0.5 cm^2^, which corresponds to an effective electrode area of about 0.05 cm^2^ (ca. 10% porosity). For comparison, CVs were also recorded using a Au macroelectrode, having an electroactive area of 0.4 cm^2^.

Morphological analysis was performed using a field emission scanning electron microscope (SEM) (Jeol JSM 7401 F). Energy-dispersive X-ray (EDX) spectra were recorded in the SEM using a Bruker spectrometer applying an acceleration voltage of 20 kV and analysed by a built-in software (Quantax). Cu TEM grids (Plano GmbH) served as substrates.

## Results and Discussion

**Figure 1:**
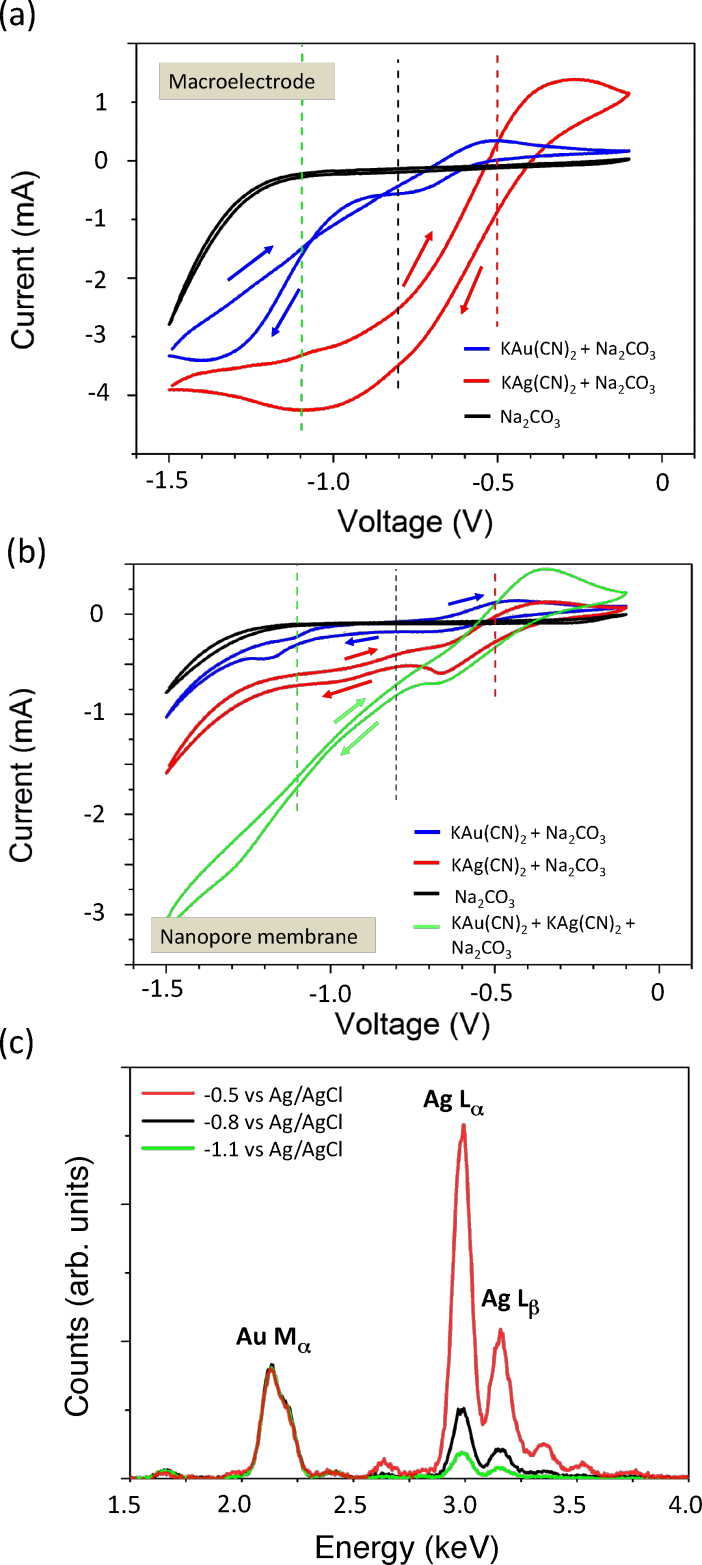
CVs using an electrolyte containing 50 mM KAu(CN)_2_ (blue line), an electrolyte containing 50 mM KAg(CN)_2_ (red line) and a solution containing only 0.25 M Na_2_CO_3_ (black line) using as working electrode (a) a gold wire and (b) a nanopore membrane with 10^9^ pores/cm^2^ and sputtered Au on the bottom membrane side. The green curve in Figure 1b corresponds to a solution containing 50 mM KAuCN_2_ and 20 mM KAgCN_2_. (c) Three representative EDX spectra of bundles of nanowires corresponding to different arrays deposited at −0.5 V (red line), −0.8V (black line), and −1.1 V (green line). The spectra are normalized to the energy of the Au Mα peak.

To synthesise segmented AuAg alloy nanowires by pulsed electrodeposition using a single-bath electrolyte, we studied electrolyte and voltage characteristics by cyclic voltammetry. Figure 1a shows a CV of an electrolyte containing 50 mM KAu(CN)_2_ (blue line) and a second one of an electrolyte containing 50 mM KAg(CN)_2_ (red line). In addition, also the curve of a solution containing only 0.25 M Na_2_CO_3_ (black line) was measured. In all cases a Au rod served as working electrode. This Au rod is chosen, since also during nanowire deposition, we use a Au layer of sputtered Au on the membrane rear side as working electrode. During the process, the voltage is ramped from −0.1 to −1.5 V and the current flowing in the cell is recorded. When approaching −1.5 V the ramping is inverted.

As expected the black curve does not reveal any characteristic peaks. The strong current increase at high negative potentials, which can also be seen in the red and the blue curve, is assigned to hydrogen evolution. The blue and the red CVs reveal that the current onset for the reduction of 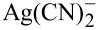 ions is initiated at potentials closer to 0 V than for the 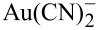 ions (Figure 1a). Accordingly, the reduction peak, indicating diffusion limited deposition, for the Au electrolyte is approached at −1.4 V. In the CV of the Ag electrolyte a peak at lower (less negative) potential of −1.1 V can be seen. While the equilibrium potential for Au^+^/Au is more positive than for Ag^+^/Ag, it is known that the equilibrium potential for the 
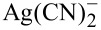
/Ag couple is more positive than that of the 
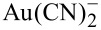
/Ag couple [51]. Thus, it is possible that Au atoms from the Au working electrode are oxidised during the preheating to 60 °C for 30 min and displaced by Ag. Therefore, the reduction peak in the CV of the Ag electrolyte might result not only from Ag deposition but also from Au deposition. For the blue curve corresponding to the Au electrolyte an additional plateau is found at around −0.75 V, which is assigned to a different deposition route of 
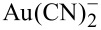
 ions. While in the diffusion-limited regime direct reduction of the 
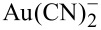
 ions occurs, it is known that at small overpotentials the deposition of Au proceeds through the absorbance of 
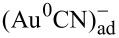
 intermediates on the electrode. [51]

Figure 1b shows four CVs recorded using a Au layer deposited on the bottom side of a track-etched membrane with 10^9^ pores/cm^2^ as working electrode. This Au backlayer on the membrane was prepared by sputtering of Au and electrodeposition of Au from a sulfide-based electrolyte (Metakem, pH 7.5) at −0.7 V in a two-electrode arrangement with a Au wire as anode. Figure 1b displays the resulting CVs for the same three electrolytes as in Figure 1a. In addition, the green curve corresponds to a CV for an electrolyte consisting of 50 mM KAu(CN)_2_ and 20 mM KAg(CN)_2_. For the CV of Au we find the diffusion limited peak at −1.2 V, this means at a slightly lower (less negative) potential than for the macroelectrode. This small shift to lower (less negative) potentials is also observed for the additional plateau at around −0.67 V in the CV of Au and can explained by the altered mass transport between a macroscopic rod-shaped electrode and the recessed electrode geometry. For the Ag electrolyte, we find a reduction peak at −0.68 V and a further current increase at higher (more negative) potential. Both, might be related to the deposition of 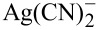 ions and additional 
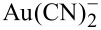
 ions, originating from the Au backlayer as in the case of the macroelectrode. It is obvious that 
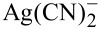
 ions are reduced at lower potential than 
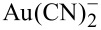
 ions, which is in accordance with the result for the macroelectrode. We find that the deposition in the membrane of Au starts at about −0.4 V and for Ag at about −0.2 V. In addition, the CV of the mixed AuAg electrolyte shows, similar to the CV for the Ag electrolyte, a plateau at about −0.7 V followed by a further current increase. The current flow is much higher due to the higher concentration of the electrolyte.

Based on these CVs, we suggest that Ag-rich nanowires can be electrodeposited with a slow rate, as needed for a well-controlled process, at low potentials close to −0.5 V. To grow Au-rich nanowires higher negative potentials need to be applied. The concentration of KAu(CN)_2_ in the electrolyte is increased compared to the KAg(CN)_2_ concentration to counteract the co-deposition of Ag at these more negative potentials.

To investigate the composition of AuAg alloy nanowires as a function of the deposition voltage, we deposited nanowire arrays at three different voltages, namely −0.5 V, −0.8 V, and −1.1 V using an electrolyte containing 20 mM KAg(CN)_2_ and 50 mM KAu(CN)_2_. The corresponding potentiostatic deposition curves at the three different voltages are shown in Supporting Information File 1 in Figure S2 together with the deposition curves using a second electrolyte with an identical Au and Ag ratio in the electrolyte. Afterwards we dissolved the polymer membrane, and transferred the wires onto TEM grids (Plano). EDX-in-SEM spectra were measured on bundles of the resulting wires. Extracts of the obtained EDX spectra are shown in Figure 1c, depicting the Lα peak (2.984 keV) and the Mα peak of Au (2.120 keV). All spectra are normalized to the height of the Mα peak of Au. The quantitative analysis reveals for the nanowires deposited at −1.1 V (green line), −0.8 V (black line), and -0.5 V (red line) compositions of about Au_60_Ag_40_, Au_40_Ag_60_, and Au_15_Ag_85_, respectively. This demonstrates that it is possible to vary the AuAg composition in the wires in a controlled manner only by varying the potential within 0.6 V.

**Figure 2:**
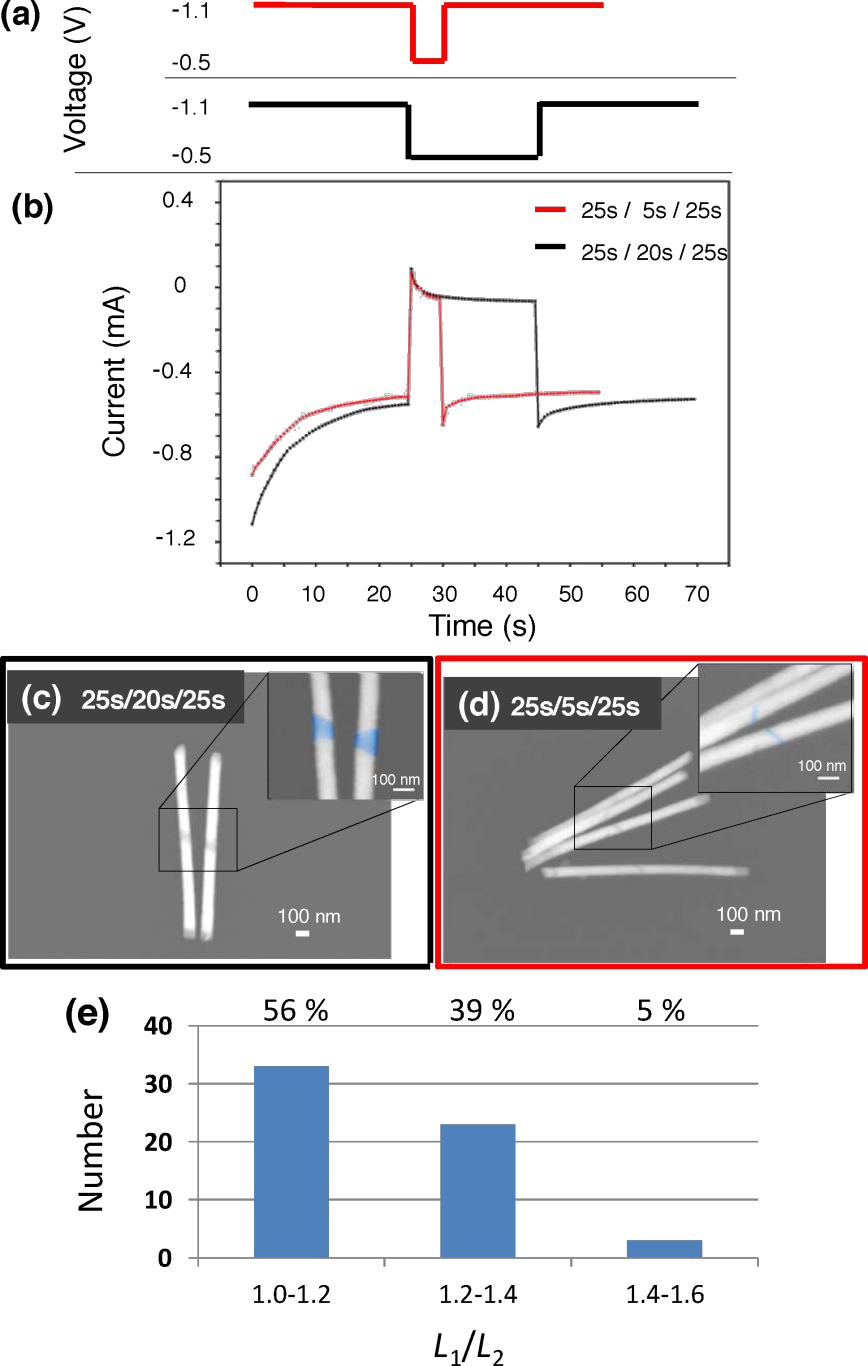
(a) Two pulse sequences applied for the electrodeposition of two arrays of segmented nanowires. The duration of the first and the third pulse is hold constant at 25 s, while the duration of the middle pulse is in the black curve 20 s and in the red curve 5 s and (b) corresponding current-versus-times curves recorded during the deposition of the nanowires. (c) and (d) SEM images of the resulting segmented wires. Image (c) corresponds to the middle pulse of 20 s and (d) to a pulse of 5 s. (e) Histogram displaying the distribution of the ratio between the length of the two Au-rich segments of the wires.

We applied these results to fabricate segmented Au-rich/Ag-rich/Au-rich nanowires with tailored composition along its axis using pulsed deposition. We choose for the synthesis a deposition voltage of *U*_1_ = −1.1 V for the Au-rich segments, and *U*_2_ = −0.5 V to create a Ag-rich middle segment. Figure 2a shows two representative pulse sequences together with the corresponding current-vs-time curves in Figure 2b. During the first and third pulse *U*_1_ is applied. Both pulses have the same duration, in this case 25 s. In between these two pulses the voltage is switched to *U*_2_. The duration of this second pulse is 5 s and 20 s for the red and the black curves, respectively.

The current-vs-time curves display a strong decrease of the absolute value of the current over about 20 s. After that, a constant cathodic current value is approached for the last five seconds of the first pulse. The decrease is due to the reduction of ions in the vicinity of the cathode and the formation of a depletion zone growing into the bulk solution. An approximately constant current is flowing during the growth of cylindrical wires. When the voltage is switched to *U*_2_ an immediate change to an anodic current is recorded, followed by a further decrease of the absolute current value to a constant small cathodic current. The anodic current is attributed to the capacitance effect [52–53] as well as to exchange displacement, which takes place since the oxidation of Au atoms occurs at similar potentials as the reduction of the 
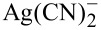
 ions as it can be seen in the CV in Figure 1b. Once enough 
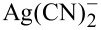
 ions are reduced, the dissolution of Au atoms is most probably suppressed, resulting in a constant current flow, during the growth of the Ag-rich segments. When the voltage is switched back to −1.1 V, the current decreases rapidly again to a high negative value, followed by a slight increase up to a constant value, which is approximately the same as for the first pulse. We measure a slight difference between the current values for the two membranes that we attribute to small differences in the number of channels and in the filling rate. A similar shape of the current-vs-time curves has previously been reported for the growth of segmented CuCo nanowires in anodized alumina templates [31,53–54].

**Figure 3:**
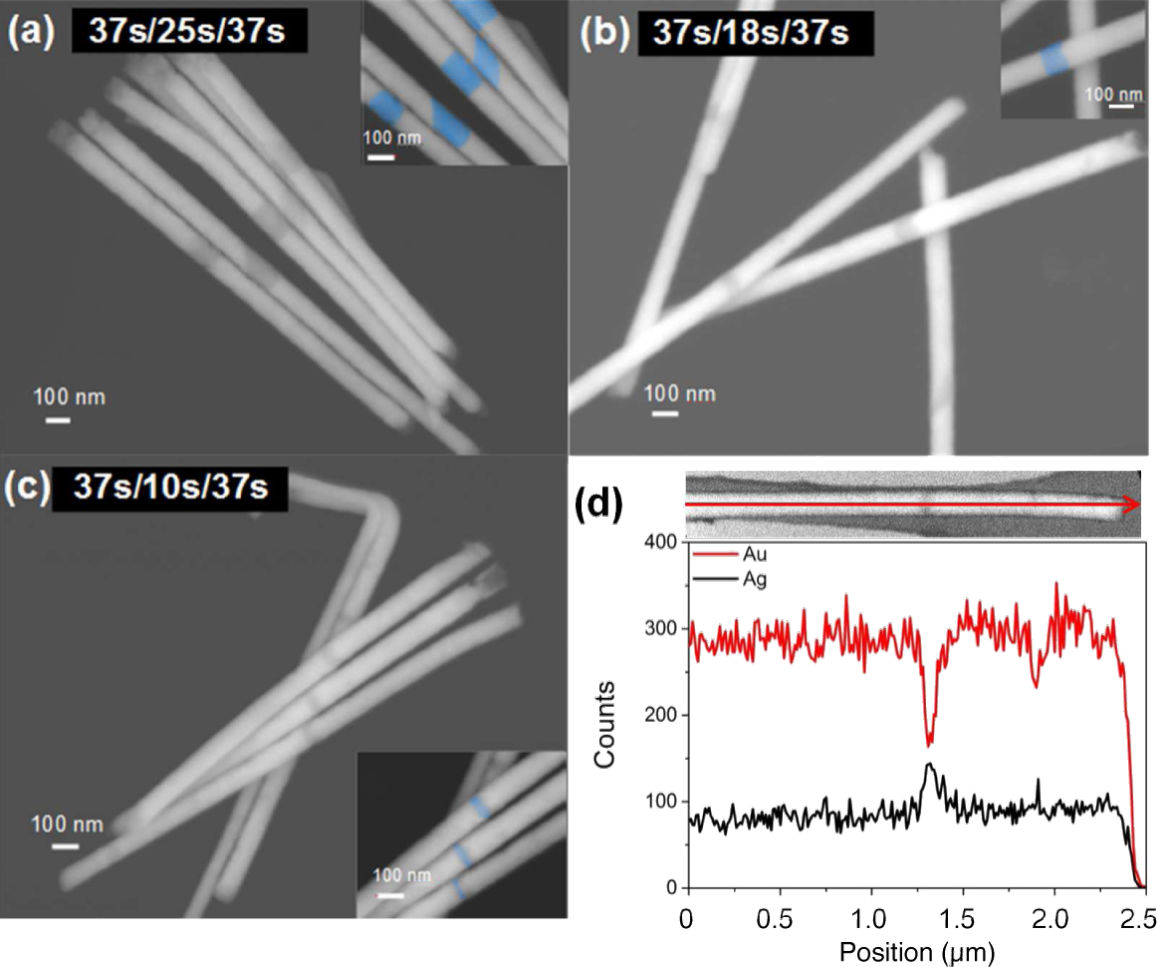
SEM images of Au_60_Ag_40_/Au_15_Ag_85_/Au_60_Ag_40_ nanowires similar to Figure 2. The length of the middle pulse is decreased form 25 s (a), to 18 s (b) and 10 s (c). In the insets the Ag-rich segments are marked in blue. (d) EDX scan along a segmented nanowire. The segmented wire is shown in the SEM image on top of the scan. The red and black lines depict the count numbers measured for the Au-Mα peak and for the Ag-Lα peak, respectively.

**Figure 4:**
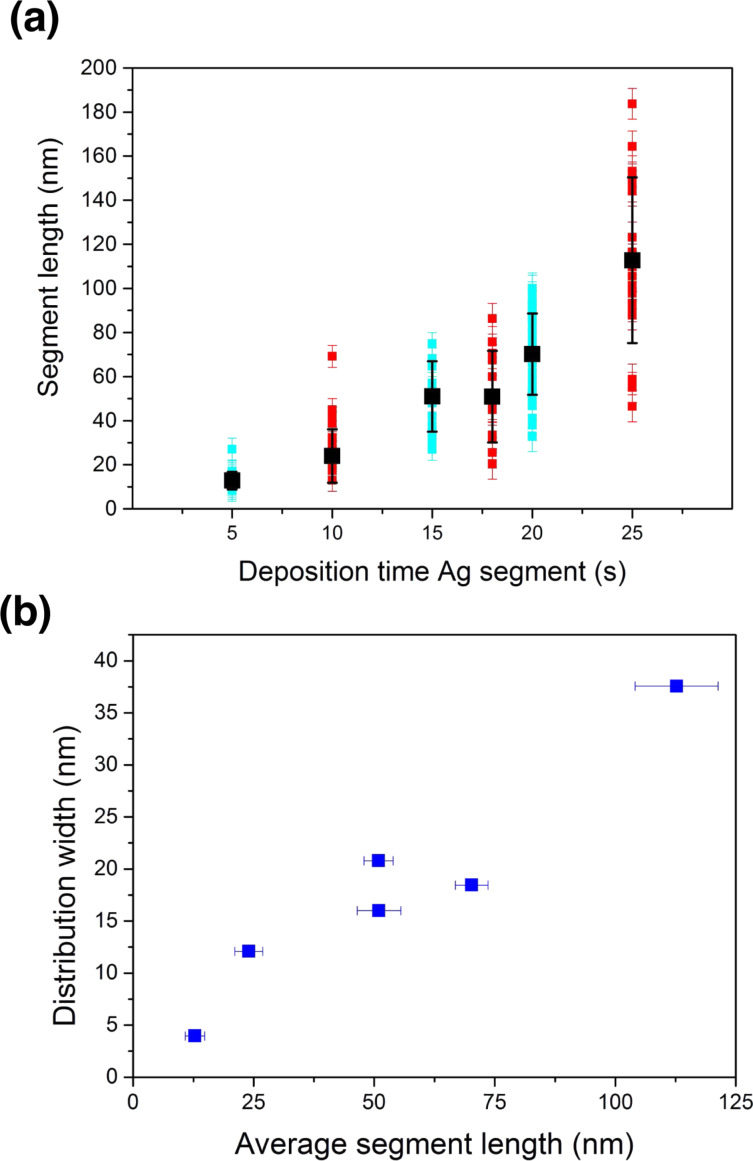
(a) Segment length of the silver segment versus pulse length for wires corresponding to six different nanowire arrays. Cyan blue values correspond to arrays with 25 s deposition time for the Au-rich segments, red values to 37 s deposition time. The black values reveal the average value for each array with its standard deviation. (b) Distribution width given by the standard deviation for the silver segments versus average value of segment length.

**Figure 5:**
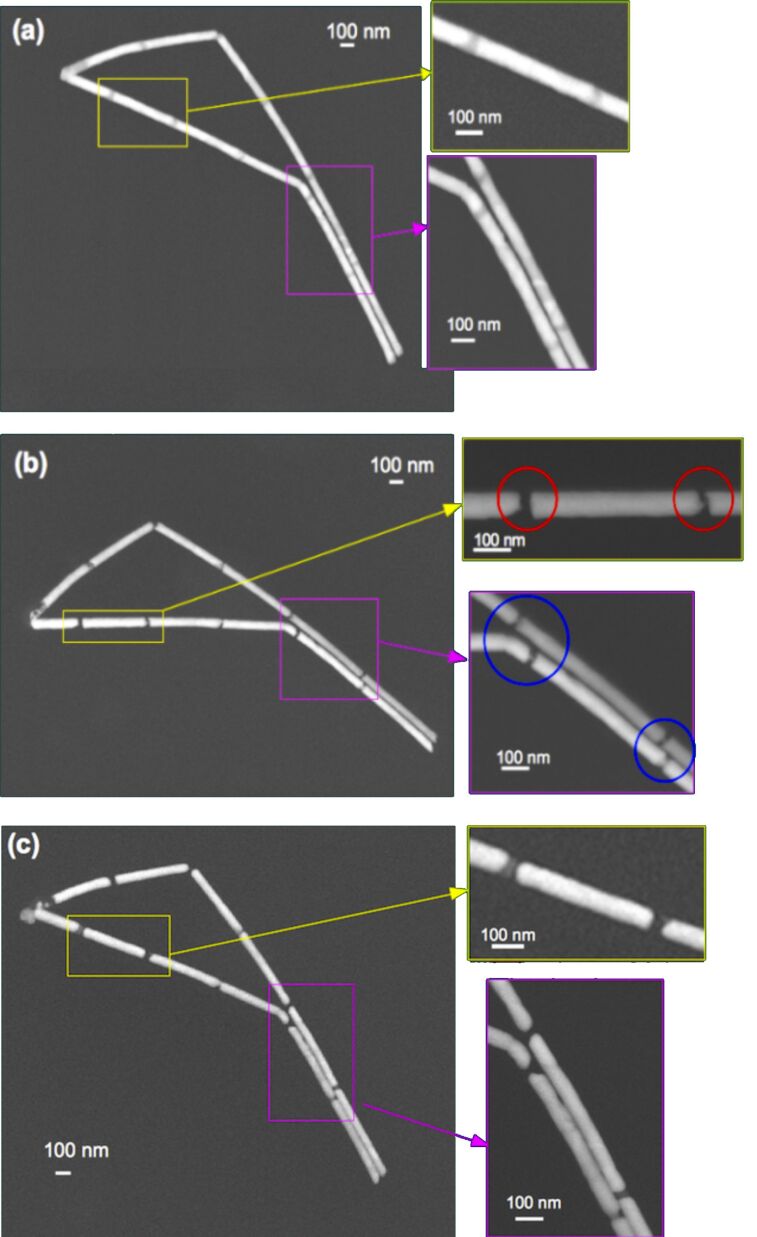
(a) SEM image of two segmented nanowires obtained from a pulse sequence of 6 pulses at −1.1 V for 25 s and 6 pulses at − 0.5 V with a duration of 15 s. (b) The same two nanowires after treatment with nitric acid. (c) Nanowires after nitric acid treatment and annealing at 300 °C for 30 min.

The SEM images in Figure 2c and Figure 2d visualize the segmented nanowires resulting from the deposition process in Figure 2a and Figure 2b. The length of the middle pulse is reduced from 20 s (Figure 2c) to 5 s (Figure 2d). The three different segments with relatively sharp interfaces are displayed by using backscattered electrons. In the details of the figure the Ag-rich segments are coloured in blue to better distinguish them from the Au-rich segments. As expected, the representative SEM images reveal a smaller Ag-rich segment for the short 5 s middle pulse (d), than for the pulse with duration 20 s (c). Figure S3 in the supporting information shows a TEM image and a high-resolution TEM image of one of the wires corresponding to the same array as the wires in Figure 2d. The figure reveals the polycrystalline structure of our wires. To investigate the symmetry of the created nanostructures, the histogram in Figure 2e shows for about 60 nanowires the ratio between the lengths *L*_1_ and *L*_2_ of the two Au-rich segments corresponding to one segmented wire. The histogram shows that 56% of the wires exhibit a length ratio between 1.0 and 1.2. Very unsymmetrical structures having a ratio higher than 1.4 are rarely observed. The average length of both Au-rich segments was estimated to be 700 ± 100 nm.

Figure 3a–c show SEM images of bundles of nanowires deposited with a similar three-pulse sequence as in Figure 2a. Compared to Figure 2, the length of the pulse at −1.1 V is increased to 37 s. The length of the middle pulse at *U* = −0.5 V is in (a) 25 s, in (b) 18 s and in (c) 10 s. The figure visualizes that the Au-rich segments are longer compared to the segments in Figure 2. Analysing 26 wires, the average length of both Au-rich segments was estimated to be 1000 ± 100 nm and 65% of the wires reveal a length ratio between 1.0 and 1.2. In agreement with the results shown previously in Figure 2, Figure 3a–c evidences again that the length of the Ag-rich segment is controlled by the pulse duration. Figure 3d displays an EDX-in-SEM scan along one of the nanowires corresponding to a sample prepared from a pulse sequence of 37 s at −1.1 V and 12 s at −0.5 V. The Ag Lα line, revealing increased count rates at the position of the dark segment in the middle of the wire, confirms position and length of the Ag-rich segment.

To investigate the segment length of the Ag-rich segments as a function of the pulse duration, we determined the length of 15–30 segments for different samples by SEM analysis (Figure 4a). Blue and red symbols distinguish between samples with pulse duration of 25 s (blue) and 37 s (red) for the Au-rich segment, respectively. The black symbols are the mean value from the measured segment length with standard deviation. The length of each Ag-rich segment is defined as the shortest distance between two Au-rich segments. The determination of the length distribution is challenging due to the different crystal orientations. In addition, only few selected wires could be measured for each sample. As expected, the mean value becomes larger with increasing deposition time, independent of the pulse length of the Au-rich segments. Figure 4b depicts the distribution width, represented by the standard deviation, versus the average value of the Ag-rich segment length. The increase in distribution width is evident. Similar behaviour is also observed for the electrodeposition of thin films where the effect is called kinetic surface roughening [55–56].

These Au/Ag/Au nanowires constitute excellent platforms for the fabrication of small nanogaps, by selective dissolution of the Ag segment. The method named “on-wire lithography” has been reported previously for wires of different noble metals [38]. In these cases, segmented nanowires were created by using different electrolytes for Au and Ag and by exchanging the electrolyte after the deposition of each segment. Our nanowires, in turn, are deposited from a single-bath electrolyte, which could influence the dissolution process and thus the morphology of the resulting gaps. Selective dissolution of the Ag-rich segments results in well-controllable gaps.

Figure 5 displays SEM images of the same two wires with diameters of about 65 nm consisting of six Au-rich and six Ag-rich segments (a) before and (b) after the nitric acid treatment. For dissolution, the wires deposited on a Si wafer were immersed for three hours into concentrated nitric acid and subsequently cleaned with deionized water. It is remarkable that the nanowires are not displaced during the nitric acid treatment. This is very important for the preparation of defined gap sizes and it furthermore enables implementation of this preparation method at larger scale applications, for example, using well-aligned arrays of nanostructures.

The insets of Figure 5a and Figure 5b clearly confirm that exclusively the Ag-rich segments are dissolved, while Au-rich segments remain intact. We find that two of the ten Ag-rich segments are completely transferred into nanogaps, while for the others, small junctions connecting two segments are visible. For some of them, it is very difficult to decide if a gap is created or if metal connects two segments. We think that the connections are due to the small amount of Au that was initially in the Ag-rich segment. The gaps found in Figure 2 have sizes of about 30 and 15 nm. The smallest gaps that we were able to create from samples with a duration of the middle pulse of 10 s had sizes of about 5 to 10 nm. Exemplarily, a TEM and a STEM in SEM image of such small gaps are shown in Figure S4 of Supporting Information File 1.

It is interesting that the morphology of the Au-rich segments is maintained, although they contain a considerable amount of Ag. By EDX analysis, we find that the Ag content in these segments is not changed through the nitric acid treatment. This effect is ascribed to dealloying in AuAg alloy structures taking place through layer-by-layer dissolution of Ag atoms and diffusion of Au atoms onto the surface [57–58]. Due to a higher Au concentration in the Au-rich segments, a Au passivation layer on the surface is quickly formed, prohibiting changes of morphology and composition. This agrees with results in [59], where morphology changes of nitric acid treated Au*_x_*Ag_1_*_−x_* is only found for a gold content of *x* ≤ 40.

In addition, we studied how moderate annealing influences shape and size of the gaps formed during the nitric acid treatment. The nanogaps were therefore annealed at 300 °C for 30 min with a heating rate of 9 °C/min. After the annealing process, seven nanogaps are visible in Figure 5c. This means that by the influence of the annealing in several cases the small junctions, connecting two Au-rich segments, could be removed. The ends of the Au-rich segments are rounded compared to the unheated segments and the gap sizes are in most of the cases increased. Rounding of the nanowire ends and removal of small bridges can be explained by surface diffusions of the atoms and the lower surface energy of spherical surfaces compared to cylindrical structures [60].

## Conclusion

In conclusion, we demonstrated the electrodeposition of segmented AuAg alloy nanowires with controlled dimensions by pulsed electrochemical deposition. The Au:Ag concentration is governed by the electrolyte and by the voltage applied during deposition. Cyclic voltammetry of Au and Ag deposition in ion-track etched membranes reveals that the current onset for deposition of Ag occurs at lower potentials than of Au. The fabrication of Ag-rich segments thus requires lower potentials than Au-rich segments. The length of Au- and the Ag-rich segments is controlled by the pulse duration. By dissolving Ag-rich segments deposited between two Au-rich segments, nanowires separated by small gaps are created. This method has previously been reported for segmented nanowires prepared by potentiostatic deposition and by exchanging the electrolyte after each segment. The process is termed on-wire lithography [38]. We have tested if this method is also applicable to our segmented wires prepared by pulsed deposition. We find that the characteristics such as gap sizes and shapes are defined by the Au- and Ag-rich segments. However, only a small number of Ag-rich segments are transformed into gaps. For the majority of the segments, small metallic interconnects remain between the segments. We attribute this to the small amount of Au atoms that are present in the Ag-rich segments. The process has been improved by annealing the wires to moderate temperatures. This allows one to increase the number of created gaps, and also modifies the shape of the remaining segments to more rounded ends. These nanowires separated by small gaps or connected by small junction are excellent candidates as model systems for plasmonic characterizations with EELS-TEM [45,61] and surface enhanced infrared spectroscopy measurements.

## Supporting Information

File 1 Supporting Information shows additional figures on the electrodeposition of segmented nanowires and gaps. Figure S1 depicts a typical ion-track membrane. Figure S2 shows the electrodeposition curves for two different electrolytes together with EDX results of the wires. Figure S3 shows TEM analysis of a segmented nanowire and Figure S4 presents TEM and SEM images of small nanogaps.
