# Supplementary material for: Growth and morphological analysis of segmented AuAg alloy nanowires created by pulsed electrodeposition in ion-track etched membranes
Source: Beilstein J Nanotechnol. 2015 Jun 8;6:1272–80. doi: 10.3762/bjnano.6.131 (PMC4505191; doi:10.3762/bjnano.6.131)
Supplement: File 1 — Supporting Information shows additional figures on the electrodeposition of segmented nanowires and gaps. Figure S1 depicts a typical ion-track membrane. Figure S2 shows the electrodeposition curves for two different electrolytes together with EDX results of the wires. Figure S3 shows TEM analysis of a segmented nanowire and Figure S4 presents TEM and SEM images of small nanogaps. [file Beilstein_J_Nanotechnol-06-1272-s001.pdf]

Supporting Information

for

Growth and morphological analysis of segmented AuAg alloy nanowires created by pulsed electrodeposition in ion-track etched membranes

Ina Schubert[*1], Loic Burr[1,2], Christina Trautmann[1,2] and Maria Eugenia Toimil-Molares[1]

Address: [1]Materials Research Department, GSI Helmholtzzentrum für Schwerionenforschung GmbH, Darmstadt, Germany and [2]Department of Materials- and Geo-Science, Technische Universität Darmstadt, Darmstadt, Germany

Email: Ina Schubert* - i.schubert@gsi.de

* Corresponding author

Additional experimental data

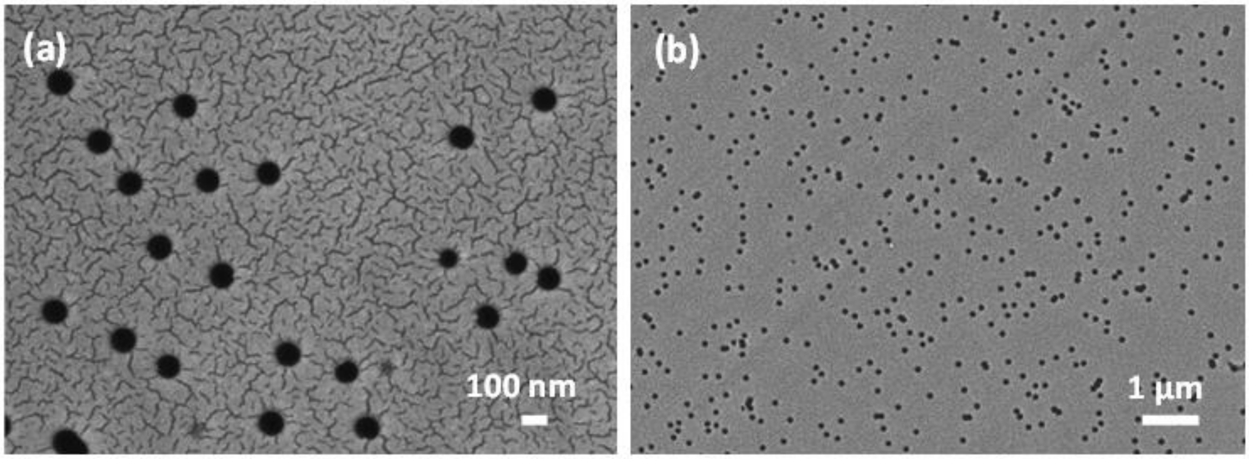

Figure S1: SEM image of a typical ion-track membrane with 10^9 ions/cm^2 with (a) high and (b) low magnification. The polymer foil is etched for 5 min after the ion irradiation.

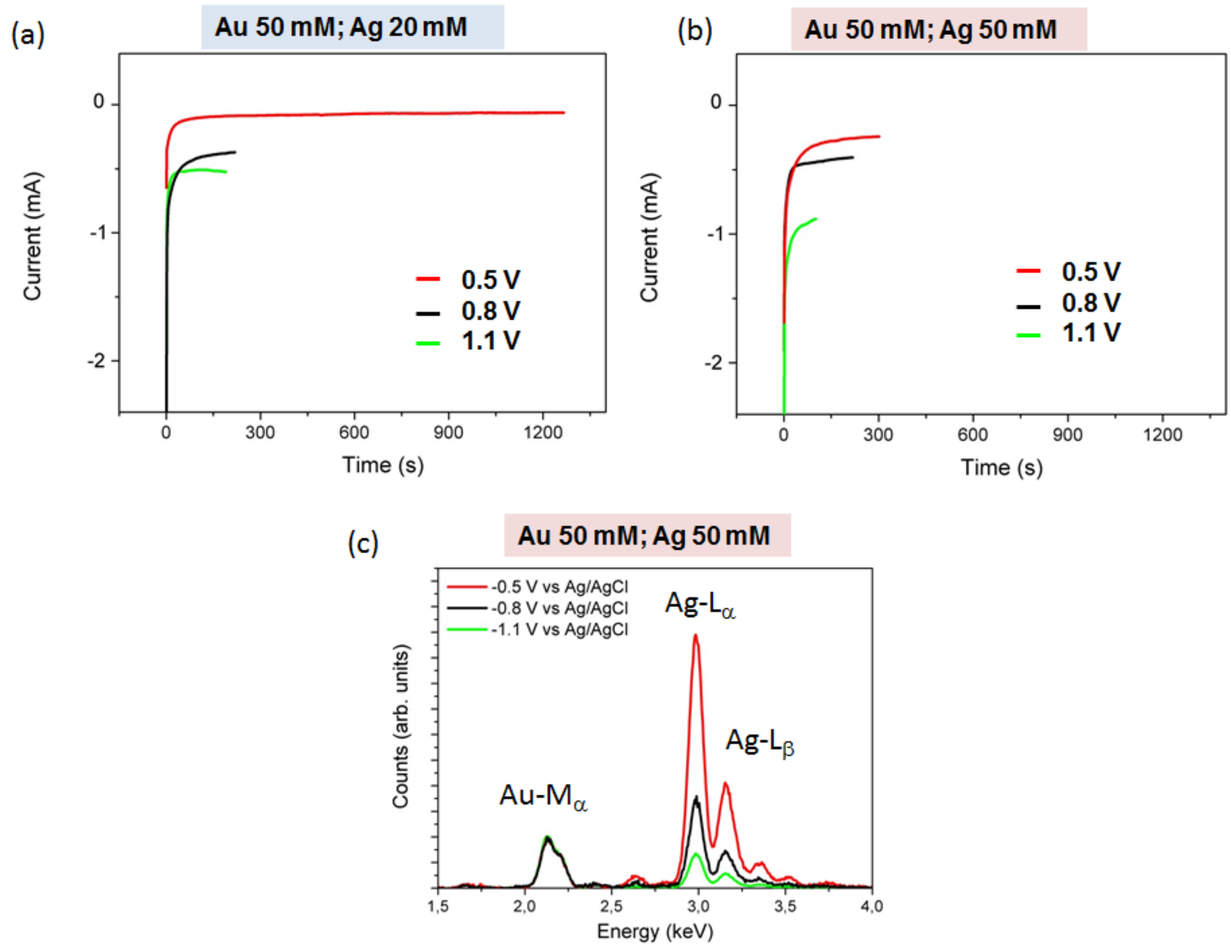

Figure S2: Potentiostatic current versus time curves for (a) an electrolyte containing 50 mM $KAu(CN)_2$ and 20 mM $KAg(CN)_2$ and (b) an electrolyte consisting of 50 mM $KAu(CN)_2$ and 50 mM $KAg(CN)_2$. All deposition parameters are given in the experimental section. In both cases three different voltages, being −0.5 V (red line), −0.8 V (black line) and −1.1 V (green line) were applied between the Ag/AgCl reference electrode and working electrode. In (c) the EDX spectra of bundles of nanowires corresponding to the three deposition curves in (b) are presented.

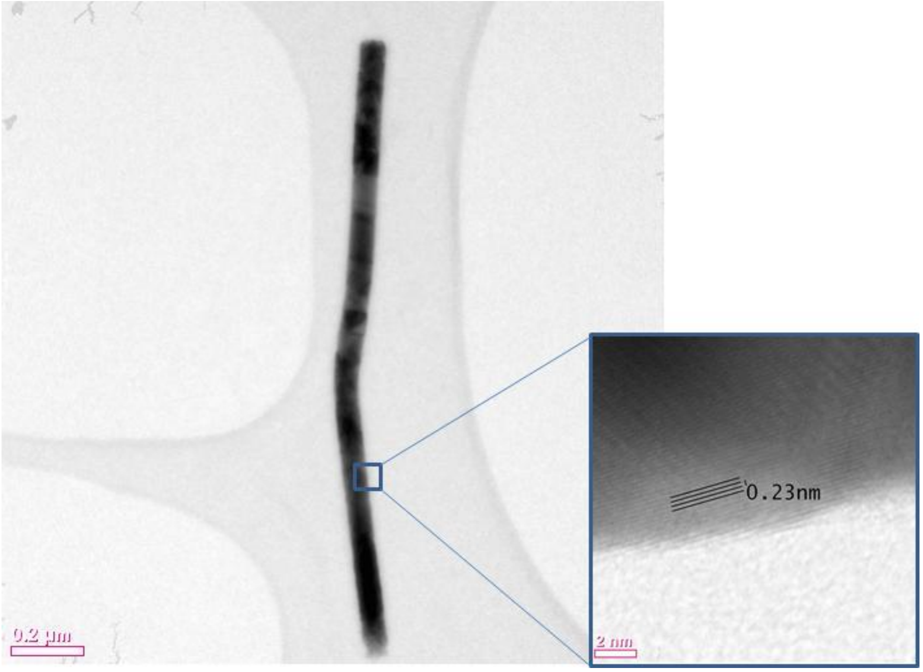

Figure S3: TEM image together with a high-resolution TEM image of a segmented nanowire showing that the segmented nanowires are polycrystalline. The wire was prepared using the same deposition parameters as given in the experimental section. A pulse sequence of −1.1V/−0.5V/−1.1 V was applied. The corresponding pulse durations for the two −1.1 V pulses were 25 s and for the −0.5 V pulse it was 5 s.

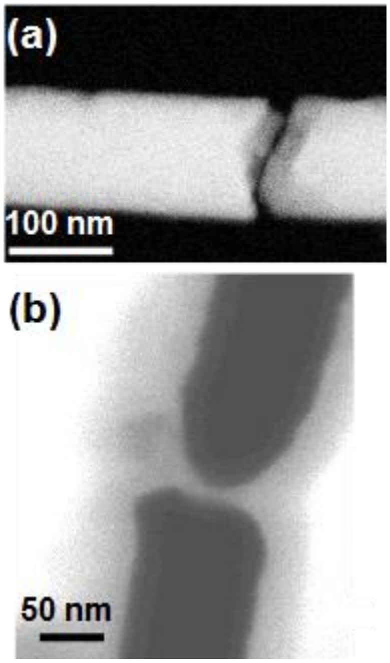

Figure S4: (a) TEM and (b) STEM in SEM image of very small gaps of about 5–10 nm prepared from pulsed deposition using a single-bath electrolyte. All parameters are given in the experimental section of the main manuscript. In both cases the pulse length for the deposition of the middle Ag-rich segment (deposited at −0.5 V vs Ag/AgCl) was 10 s.
